# One Hundred Explicit Definitions of Potentially Inappropriate Prescriptions of Antibiotics in Hospitalized Older Patients: The Results of an Expert Consensus Study

**DOI:** 10.3390/antibiotics13030283

**Published:** 2024-03-20

**Authors:** Nicolas Baclet, Emmanuel Forestier, Gaëtan Gavazzi, Claire Roubaud-Baudron, Vincent Hiernard, Rozenn Hequette-Ruz, Serge Alfandari, Hugues Aumaître, Elisabeth Botelho-Nevers, Pauline Caraux-Paz, Alexandre Charmillon, Sylvain Diamantis, Thibaut Fraisse, Pierre Gazeau, Maxime Hentzien, Jean-Philippe Lanoix, Marc Paccalin, Alain Putot, Yvon Ruch, Eric Senneville, Jean-Baptiste Beuscart

**Affiliations:** 1 CHU Lille, University of Lille, F-59000 Lille, France; 2 Groupe Hospitalier de l’Institut Catholique (GHICL), Service de Maladies Infectieuses, Université Catholique de Lille, F-59160 Lille, France; 3 Service de Maladies Infectieuses, Centre Hospitalier Métropole Savoie, F-73000 Chambéry, France; 4 Clinique Universitaire de Médecine Gériatrique, Centre Hospitalier Universitaire de Grenoble-Alpes, GREPI EA7408 Université Grenoble-Alpes, F-38000 Grenoble, France; 5 CHU Bordeaux, Pôle de Gérontologie Clinique, University of Bordeaux, INSERM 1312 BRIC, F-33000 Bordeaux, France; 6 Service de Maladies Infectieuses, CH Roubaix, F-59056 Roubaix, France; 7 Service Universitaire de Maladies Infectieuses et Tropicales, Hôpital Gustave Dron, F-59200 Tourcoing, France; 8 Service de Maladies Infectieuses et Tropicales, Centre Hospitalier de Perpignan, F-66000 Perpignan, France; 9 Infectious Diseases Department, University Hospital of Saint-Etienne, GIMAP (EA 3064), F-42055 Cedex 02 Saint-Etienne, France; 10 Faculty of Medicine of Saint-Etienne, University of Saint-Etienne, F-42023 Cedex 02 Saint-Etienne, France; 11 Faculty of Medicine, University of Lyon, F-69000 Lyon, France; 12 Service de Maladies Infectieuses et Tropicales, Hôpital Intercommunal de Villeneuve-Saint-Georges, F-94190 Villeneuve-Saint-Georges, France; 13 CHRU-Nancy, Infectious Diseases Department, F-54000 Nancy, France; 14 Grand Est Antibiotic Stewardship Network Coordinator, AntibioEst, F-54000 Nancy, France; 15 Service de Maladies Infectieuses et Tropicales, Hôpital de Melun, F-77000 Melun, France; 16 Unité de Recherche DYNAMIC, Université Paris-Est Créteil, F-94000 Créteil, France; 17 Court Séjour Gériatrique Aigu, Centre Hospitalier Alès-Cévennes, F-30100 Alès, France; 18 Service des Maladies Infectieuses et Tropicales, CHRU de Brest, F-29609 Brest Cedex, France; 19 Department of Internal Medicine, Infectious Diseases and Clinical Immunology, University Hospital of Reims, F-51100 Reims, France; 20 EA3797-Viellissement Fragilité, Reims Champagne Ardennes University, F-51100 Reims, France; 21 AGIR UR 4294, University Picardie Jules Verne, F-80000 Amiens, France; 22 Department of Infectious Diseases, Amiens University Hospital, F-80000 Amiens, France; 23 Pôle de Gériatrie, CHU Poitiers, Université Poitiers, F-86000 Poitiers, France; 24 Centre d’Investigation Clinique CIC 1402, INSERM CHU Poitiers, Université Poitiers, F-86000 Poitiers, France; 25 Médecine Interne et Maladies Infectieuses, Hôpitaux du Pays du Mont Blanc, F-74700 Sallanches, France; 26 Physiopathologie et Epidémiologie Cérébro-Cardiovasculaires, Université de Bourgogne, F-21000 Dijon, France; 27 Department of Infectious Diseases, Strasbourg University Hospital, F-67000 Strasbourg, France

**Keywords:** antimicrobial resistance, elderly, inappropriate prescription, antibiotic stewardship, hospital setting

## Abstract

Background: In geriatrics, explicit criteria for potentially inappropriate prescriptions (PIPs) are useful for optimizing drug use. Objective: To produce an expert consensus on explicit definitions of antibiotic-PIPs for hospitalized older patients. Methods: We conducted a Delphi survey involving French experts on antibiotic stewardship in hospital settings. During the survey’s rounds, the experts gave their opinion on each explicit definition, and could suggest new definitions. Definitions with a 1-to-9 Likert score of between 7 and 9 from at least 75% of the participants were adopted. The results were discussed during consensus meetings after each round. Results: Of the 155 invited experts, 128 (82.6%) participated in the whole survey: 59 (46%) infectious diseases specialists, 45 (35%) geriatricians, and 24 (19%) other specialists. In Round 1, 65 explicit definitions were adopted and 21 new definitions were suggested. In Round 2, 35 other explicit definitions were adopted. The results were validated during consensus meetings (with 44 participants after Round 1, and 54 after Round 2). Conclusions: The present study is the first to have provided a list of explicit definitions of potentially inappropriate antibiotic prescriptions for hospitalized older patients. It might help to disseminate key messages to prescribers and reduce inappropriate prescriptions of antibiotics.

## 1. Introduction

The development of antimicrobial resistance remains a major public health issue [1] and is promoted by the inappropriate use of antibiotics [2,3] (defined as under-use, over-use, incorrect choice, or incorrect use with regard to the dose level, administration route, duration of treatment, etc.) [4]. Many national and international action plans have been developed to reduce inappropriate antibiotic prescribing and to combat antibiotic resistance [5,6,7]. The appropriateness or inappropriateness of antibiotic prescriptions is usually assessed by an expert with regard to clinical practice guidelines and the individual patient’s situation; this is a so-called implicit approach. The problem of inappropriate prescriptions of antibiotics remains significant, despite effective interventions. This usual approach is time-consuming and resource-consuming for antibiotic stewardship teams to deal with a large number of prescriptions. The development of new tools remains useful for improving the use of antibiotics and consequently limiting the increase in antibiotic resistance. Another approach (already used in the field of geriatrics) is based on explicit criteria for potentially inappropriate prescriptions (PIPs) [8,9,10]. These explicit criteria (i) provide training tools for prescribers; (ii) allow the development of computerized tools for the automatic detection of PIPs; and (iii) might provide epidemiological data on these prescriptions [11,12]. The explicit approach could be an additional support for expert teams, for example by helping to disseminate messages to prescribers about prescriptions to be avoided, or by helping to better identify patients requiring optimization of the antibiotics prescribed. Older patients are particularly susceptible to bacterial infections and are frequently prescribed antibiotics [13,14,15]. In this population, possible atypical clinical presentations can affect the diagnosis, which contributes to inappropriate prescriptions of antibiotics [16].

The explicit approach has not yet been applied in antibiotic stewardship programs, and there are no validated criteria for PIPs of antibiotics (henceforth referred to as “antibiotic-PIPs”) [17]. The objective of the present study was to develop a list of explicit definitions of antibiotic-PIPs for hospitalized patients aged 75 or over.

## 2. Results

### 2.1. Participants

A total of 155 people were invited to participate in the study. Of these, 128 completed the entire survey (i.e., Rounds 1 and 2), giving a full participation rate of 82.6%. A total of 59 participants (46.1%) were ID specialists, 45 (35.2%) were geriatricians, and 24 (18.8%) were other specialists (18 hospital pharmacists, 3 microbiologists, 1 infection control practitioner, 1 general practitioner, and 1 neurologist). The characteristics of the study participants are summarized in Supplementary Data S3 and their geographical distribution is shown in Supplementary Data S4.

### 2.2. The Delphi Survey

**Figure 1:**
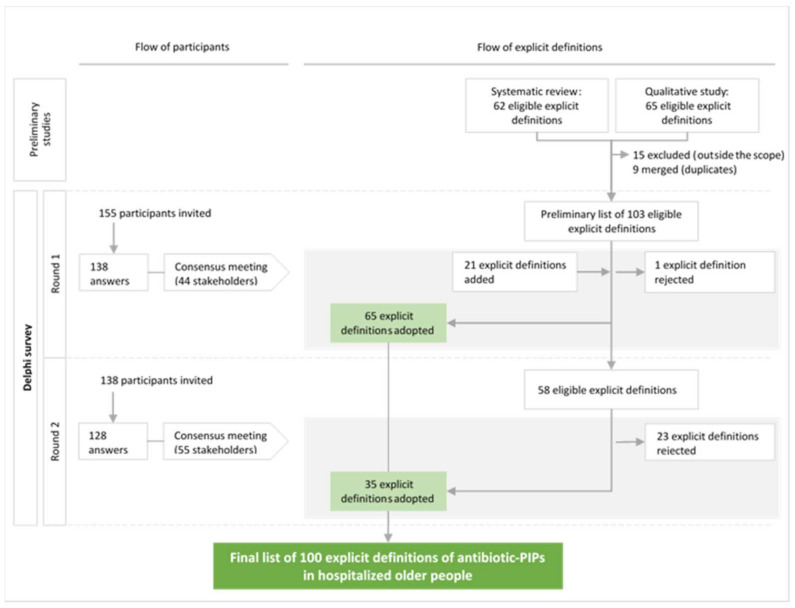
Diagram showing the flow of participants and explicit definitions of potentially inappropriate prescriptions of antibiotics (antibiotic-PIPs) during the Delphi survey and the consensus meetings.

The study flow chart for participants and explicit definitions is shown in Figure 1. The consensus meetings after Round 1 and Round 2 were attended by 44 and 55 stakeholders, respectively. At the end of Round 1, 65 of the 103 explicit definitions in the eligible list were adopted and 1 definition was rejected. The participants suggested a total of 113 new, explicit definitions of antibiotic-PIPs and several reformulations of definitions. The first consensus meeting validated the reformulation of 16 definitions and the introduction of 21 new definitions. The remaining 37 explicit definitions lacking a consensus and the 21 new definitions were submitted to Round 2 of the survey. After Round 2, 35 explicit definitions were adopted. Of the 21 new, explicit definitions suggested by the participants, 8 did not have a consensus. During the consensus meeting following Round 2, the 55 stakeholders evaluated these eight definitions and decided: (i) not to submit them to a third round, considering that they were not derived from the same standardized process as the definitions in the eligible list; (ii) to reject the five definitions if 60% or less of the experts rated them with a score of 7–9; (iii) to discuss and then vote for the three remaining definitions rated by >60% of the experts with a score of 7–9. Finally, the three remaining definitions were included (>90% of the participants voted “Yes”).

### 2.3. The Consensus List of Explicit Definitions of Antibiotic-PIPs

The Delphi survey resulted in a consensus list of 100 explicit definitions of antibiotic-PIPs for hospitalized older patients, covering 23 areas of antibiotic prescribing (Table 1). The experts specified that these explicit definitions should be applied only if the clinical presentation was non-severe and in the absence of a known drug allergy. The final list of explicit definitions of antibiotic-PIPs is given in Table 2.

## 3. Material and Methods

### 3.1. Study Design

We conducted a Delphi survey, i.e., a method frequently used to design and validate explicit criteria [18]. This sequential process collects the opinion of a panel of experts on a preliminary list of items. Each stage is called a round, in which participants fill out a questionnaire. Anonymity is a fundamental principle of the Delphi survey. Participants interact indirectly, in order to avoid the dominant influence of certain participants [19]. Our work followed expert recommendations on the reporting of Delphi studies [20,21,22,23].

### 3.2. Scope of the Study

In clinical pharmacology, PIPs can be assessed via an implicit judgment or via explicit criteria [24,25]. Explicit definitions state situations that are usually considered to be inappropriate, according to the literature or an expert consensus. In the present explicit approach, we sought to develop a consensus list of explicit definitions of antibiotic-PIPs in older patients (aged 75 or over) hospitalized in acute care units. Explicit definitions of PIPs are usually intended to limit adverse events at the individual patient level [8,9,10]. In the present work, we conceived explicit definitions as a means of combatting antimicrobial resistance on both the individual and collective scales, as presented in two preliminary studies [17,26].

### 3.3. Steering Committee

A steering committee (NB, ES, and JBB) was set up to validate the study’s methodological principles and to validate the results at each stage. The work was carried out in partnership with the GInGer (a joint study group for infections in the elderly, created by the French Infectious Diseases Society (Société de pathologie infectieuse de langue française, SPILF) and the French Gerontology and Geriatrics Society (Société Française de Gériatrie et Gérontologie, SFGG)).

### 3.4. Ethical Approval

We confirmed that the experts had agreed to participate in the study and had consented to the collection of personal data, in accordance with the European Union’s General Data Protection Regulation.

### 3.5. Preparation of the List of Eligible Explicit Definitions of Antibiotic-PIPs

The list of eligible explicit definitions of antibiotic-PIPs to be submitted to the Delphi group was prepared in two preliminary studies. Firstly, a list of 62 explicit definitions of antibiotic-PIPs was identified through a systematic review of the literature [17]. Secondly, a list of 65 explicit definitions was established in a qualitative survey [26]. The sets of explicit definitions from the two studies overlapped to some extent. We, therefore, sought to identify and merge similar definitions and to independently validate the choices made. The key steps in the preparation of this list were (i) translation of the list of definitions from the systematic literature review into French; (ii) the grouping together, merger, or reformulation of explicit definitions if necessary (performed by two researchers: NB and RHR); and (iii) validation of the list by the steering committee and external experts comprising infectious disease (ID) specialists and geriatricians. The methodology used to prepare the list of eligible explicit definitions is detailed in Supplementary Data S1. Supplementary Data S2 shows the list of eligible explicit definitions of antibiotic-PIPs.

### 3.6. The Panel of Experts for the Delphi Study

We sought a variety of opinions from ID specialists, geriatricians, and other experts in the use of antibiotics in older patients in hospitals from throughout metropolitan France, in order to take account of possible local and regional disparities in practice [27].

#### 3.6.1. Inclusion Criteria for Participants

Three groups of participants were identified, with the following target distribution: ID specialists (40%), geriatricians (40%), and other specialists involved in antibiotic stewardship in hospitals (20%). Participants had to meet at least one of the following criteria: prescriber of antibiotics, an advisory role on antibiotic therapy, membership of a hospital antibiotic committee, active membership of a learned society’s antibiotic working group, or membership of public health authority dealing with antibiotic use.

#### 3.6.2. Number of Participants

With a view to being representative and to take account of differences in expert opinion, we sought to recruit between 140 and 160 participants. We expected at least 75% of the recruited experts to participate throughout the duration of the study.

#### 3.6.3. Recruitment of Participants

The steering committee and GInGer identified 16 nationally known experts as local coordinators (EF, CBR, SA, HA, EBN, PCP, AC, SD, TF, PG, MH, JPL, MP, AP, YR, and ES). These experts were asked to (i) recruit at least three geriatricians, three ID specialists, and two other specialists meeting the inclusion criteria from within their local network; (ii) participate in the Delphi survey; (iii) participate in the consensus meetings; and (iv) help the steering committee to remind participants to fill out the questionnaire, if necessary.

### 3.7. Preparation of the Delphi Survey

#### 3.7.1. The Online Platform

The online survey was prepared on the SmartSurvey^TM^ platform (https://www.smartsurvey.com/, accessed on 12 March 2024). An introductory page presented the concepts needed to understand the study’s scope and objectives, with a synopsis, an explanatory video, and the results of the preliminary studies (i.e., an information kit). The participants were informed that their answers would be anonymous and were given information about the regulatory framework that covered the data collected. After confirming that they did not object to these conditions, the participants filled out a questionnaire collecting data on their age, sex, year of their MD/PharmD thesis, city of practice, type of hospital (general or university), specialty, antimicrobial stewardship activity, membership of an antibiotic committee, membership of a learned society’s antibiotic working group, and membership of a public health authority dealing with antibiotic use. Next, the main questionnaire presented all the explicit definitions of an antibiotic-PIP, which were scored on a Likert scale ranging from 1 (strongly disagree) to 9 (strongly agree) [28]. To avoid exhaustion bias, the various definitions were presented in random order.

#### 3.7.2. Briefing of the Participants

An individual meeting (1 h) with each local coordinator was used to present the study and the recruitment method and to explain the coordinator’s role. Next, the steering committee and the local coordinator met each participant and presented the study process (1 h). Lastly, each participant received an e-mail message containing the information kit (the one available online) and a personal link to the online survey.

### 3.8. Definition of the Consensus Criteria

Participants were asked to express their level of agreement with each explicit definition of antibiotic-PIP by rating it on the above-mentioned 1-to-9 Likert scale [28]. The objective was to adopt explicit definitions with a high level of consensus. The numerical criteria for consensus were as follows. If at least 75% of the participants gave a Likert score of between 7 and 9, the definition was adopted. If at least 75% of the participants gave a Likert score of between 1 and 3, the definition was rejected. In all other cases, no consensus was formed.

The Delphi survey method encourages discussion of the numerical results by the participants. Consensus meetings were organized at the end of each round and were attended by the local coordinators and available experts from the Delphi panel. These meetings were intended to present the results of each round, summarize the comments, and discuss contentious cases. Each proposed change in the wording of an explicit definition was discussed and voted on during the meetings, using the Wooclap application (https://www.wooclap.com/, accessed on 12 March 2024).

### 3.9. The Delphi Process

#### 3.9.1. The Rounds

In Round 1 of the survey, each participant expressed his/her level of agreement with each explicit definition on a scale of 1 to 9 and could also provide a free text comment if so wished. Participants were also allowed to suggest new, explicit definitions in this round. In Round 2, each participant again expressed his/her opinion on explicit definitions on the 1-to-9 scale. The explicit definitions of antibiotic-PIPs considered in Round 2 included (i) the explicit definitions from the eligible list (Supplementary Data S2) that did not achieve a consensus in Round 1, and (ii) the new, explicit definitions suggested in Round 1. If a participant did not reply, he/she was sent a reminder email by the investigators. Another reminder was sent by the regional coordinator, if needed.

#### 3.9.2. Analysis of Rounds and Preparation of the Consensus Meetings

The data were analyzed statistically using R software (version 4.1.2) [29]. With regard to the participants’ characteristics, qualitative variables were quoted as the frequency (percentage) per category, and quantitative variables were described as the median (range). Explicit definitions were classified as having been adopted, rejected, or lacking a consensus. Each free text comment was analyzed independently and in a standardized manner by two researchers (NB and VH), in order to identify critical comments that might prompt the reformulation of explicit definitions. Disagreements were resolved by the two researchers and then by the steering committee, if necessary. The new definitions of antibiotic-PIPs suggested by the participants in Round 1 were checked for explicitness by two researchers (NB and VH). The steering committee validated the results of the quantitative analysis and the text content analysis for submission to the consensus meeting.

#### 3.9.3. Consensus Meetings

Consensus meetings (videoconferences) were organized by the investigators at the end of each round. The investigators first chose the date with the regional coordinators (so that all of the latter could attend the meeting) and then invited all Delphi participants. The results obtained by the research team were presented and discussed after each round. The objectives of the meeting after Round 1 were to (i) adopt the explicit definitions of antibiotic-PIPs that meet the consensus criteria; (ii) validate the reformulation of certain explicit definitions; and (iii) validate the new, explicit definitions proposed in Round 1, in order to submit them to Round 2.

The consensus meeting after Round 2 notably concerned the explicit definitions from the eligible list (Supplementary Data S2); the objective was to adopt those that met the consensus criteria. Definitions that did not have a consensus were definitively rejected. This second meeting also concerned new, explicit definitions of antibiotic-PIPs suggested by the participants in Round 1. The objectives were to (i) adopt those that met the consensus criteria; and (ii) discuss those classified as lacking a consensus for protocol adaptation. These new, explicit definitions did not result from the same preparation process as the list of eligible explicit definitions (Supplementary Data S1). We used the results for the definitions from the eligible list in Rounds 1 and 2: none of the definitions with a favorable score (7–9) from 60% or less of the experts in Round 1 were adopted in Round 2. We, therefore, decided that new, explicit definitions with a favorable score (7–9) from 60% or less of the experts could be rejected directly and that definitions with a favorable score of between 60 and 75% of the experts were contentious and had to be discussed and then voted on (yes/no) for definitive adoption or exclusion.

## 4. Discussion

The present study is the first to have provided a list (n = 100) of explicit definitions of antibiotic-PIPs for hospitalized older patients. This list provides key messages for prescribers and could be used in specific computer-based tools for the detection of inappropriate situations. Application of this list might help to reduce antibiotic-PIPs and, thus, contribute to the fight against antimicrobial resistance.

### 4.1. A New Approach to Antimicrobial Stewardship

The explicit approach is already used in the field of geriatrics, where several lists of explicit criteria for inappropriate drug prescriptions have been validated [8,9,10]. The explicit criteria developed in geriatrics are intended to limit individual adverse events for older patients and have proven value in reducing inappropriate prescriptions [30] and related clinical events, such as falls, confusion, and hospital readmission [31,32].

Our work was inspired by this approach, with application in the use of antibiotics. Explicit definitions were developed with a view to limiting the development of bacterial resistance on individual and collective levels (e.g., by using broad-spectrum compounds sparingly and by limiting treatment times). These explicit definitions were designed in a very general way so that a wide range of PIPs could be detected. It should be kept in mind that explicit definitions of antibiotic-PIPs can flag up situations considered to be *potentially* inappropriate but cannot determine with certainty the inappropriateness of a given prescription. There is a degree of overlap between explicit approaches, which can provide general information, and implicit approaches, which must subsequently be validated by an expert in antibiotic prescribing. For example, the explicit definitions of antibiotic-PIPs might help to flag up situations that are potentially inappropriate and can then be reassessed by an expert. This approach might improve the detection of inappropriate prescribing situations, enable actions to be targeted more effectively, and foster the efforts of antibiotic stewardship teams.

### 4.2. Explicit Definitions of Relevance to the Fight against Antibiotic Resistance

The explicit definitions in our list corresponded to ongoing issues in antibiotic stewardship. For example, 40% of the definitions were concerned with urinary tract, respiratory tract, or skin infections—the main infections encountered in hospitalized older patients. The most commonly cited antibiotics in the definitions were those qualified as critical: fluoroquinolones, amoxicillin–clavulanic acid, cephalosporins, aminoglycosides, and carbapenems [33]. Many of the definitions were intended to limit the use of these antibiotics when the infection was documented (i.e., the infection site and/or the microorganism) and to facilitate the application of general principles when the infection was not documented, with the aim of limiting the use of broad-spectrum antibiotics. Other explicit definitions specified treatment times that should not be exceeded (e.g., for respiratory or skin infections), in accordance with recent guidelines [34,35,36]. In our systematic review [17], we showed that the published explicit definitions did not adequately cover some issues in antibiotic use. Our list provides new, explicit definitions that address the domains most commonly encountered in practice by multidisciplinary antibiotic therapy teams and the core elements of hospital antibiotic stewardship programs [37,38], such as the general principles of antibiotic use (e.g., definitions #54: It is potentially inappropriate to prescribe ertapenem as a first-line treatment; and #57: It is potentially inappropriate to prescribe antibiotics for an isolated elevation of C-reactive protein).

### 4.3. Perspectives for the Use of Explicit Definitions of Antibiotic-PIPs

The list of explicit definitions of antibiotic-PIPs could be used to provide training messages to prescribers on situations considered to be inappropriate and that should be avoided. These explicit definitions could also be integrated into computer-based decision support systems for the detection of antibiotic-PIPs [31,39]. This type of detection would provide epidemiological data on antibiotic-PIPs on different scales (e.g., a department, a hospital, etc.). This information would also facilitate audits and assessments of professional practice, in order to provide personalized messages on improving antibiotic use. Lastly, real-time detection could help multidisciplinary antibiotic stewardship teams to re-evaluate potentially inappropriate treatments in a more targeted way to increase the efficiency of interventions. Clinical validation of the definitions provided in this study could, therefore, be based on studies evaluating the validation rate between automatic detection based on explicit definitions, using a digital tool, and the opinion of an expert in infectiology. Implementation in clinical decision support systems will require a set of procedures for their use in everyday practice: translating the explicit definitions into semi-natural language, then translating them into computer language, testing the rules (technical errors, clinical relevance, effective changes to prescriptions), deploying them within a hospital structure, and measuring an outcome [40,41,42]. Studies using this explicit approach would be able to assess the effect of antibiotic therapy re-evaluation interventions, based on the detection of antibiotic-PIPs, on patient outcomes and, in the longer term, the effects on the development of bacterial resistance.

The list developed here was designed for use with older inpatients. However, many of the definitions do not appear to be limited to older adults and might be applicable to hospital patients in general (e.g., definitions #60: “It is potentially inappropriate to prescribe carbapenems for empirical treatment definitions” and #74: “It is potentially inappropriate to prescribe antibiotics for more than 7 days for pneumonia”). Some of the definitions of antibiotic-PIPs that are applicable to adults in general are necessarily applicable to older adults. Although this was not one of the present study’s objectives, it might be possible to use a subset of the definitions in younger adults—subject to expert validation.

### 4.4. Strengths and Limitations

All the steps in this work followed a robust methodology. The list of eligible explicit definitions was derived from two published preliminary studies [17,26]. Furthermore, all the steps followed a dual analysis, with validation by the steering committee and then by the participants. A large number of experts participated in the Delphi survey, with a high full-study participation rate. A large number of experts also participated in the consensus meetings after each round.

The study had some limitations. Firstly, it was carried out in France, where the epidemiology of antimicrobial resistance and challenges in antibiotic use might differ from those in other countries in Europe or worldwide. However, several aspects of our list cover universal issues such as reducing treatment times, using broad-spectrum antibiotics sparingly, and avoiding unnecessary courses of antibiotics [5,6,7]. Secondly, the Delphi survey (carried out in 2021–2022) came several years after the qualitative study (2019) and the systematic review (2017). This long time interval was due mainly to the COVID-19 pandemic, during which infectious disease experts and geriatricians were not available for participation in the study. Hence, some new data in the literature might have been overlooked, although giving the experts an opportunity to suggest new, explicit definitions and holding discussions at the consensus meetings enabled us to include some recent scientific developments in this field. The consensus criteria were modified at the second consensus meeting with regard to eight definitions from those suggested by participants (not for the definitions of the eligible list). These modifications are allowed by the Delphi method [22]. They were discussed and validated by the 55 participants at the consensus meeting, in order to take into account the fact that the new definitions suggested in Round 1 were not derived from the same standardized process as the definitions in the eligible list. Therefore, eight explicit definitions that lacked a consensus after Round 2 were not submitted to a third round. Some participants may have had conflicts of interest that could have influenced their responses. However, the potential impact on the results was mitigated by the large number of participants and the variety of specialties.

## 5. Conclusions

We produced the first ever consensus list of explicit definitions (n = 100) of antibiotic-PIPs for hospitalized older patients, by using a structured Delphi method with a large number of French experts and a high participation rate. The list provides key messages to prescribers and can be used in specific computer-based tools for detecting PIPs and for improving antibiotic stewardship. Application of this list might help to reduce antibiotic-PIPs and, thus, contribute to the fight against antimicrobial resistance.

## Figures and Tables

**Table 1:** Numbers of explicit definitions of antibiotic-PIPs in hospitalized older patients, by domain or usage.

Class/Domain	Number of Definitions (*n* = 100)
**Infection site**	**52**
Upper respiratory tract	12
Urinary tract	11
Lower respiratory tract	10
Skin and soft tissues	7
Gastrointestinal tract	5
Bones/joints	4
Dental care	2
Bloodstream	1
**Use**	**18**
Administration route	5
Dose level	4
Antibiotic combination	4
Treatment time	4
Laboratory assays	1
**General principles of antibiotic use**	**16**
All infection sites	7
Undocumented infections	5
Community-acquired infections	4
**Organisms**	**14**
*Clostridioides difficile*	4
Viruses	3
*Treponema pallidum*	2
*Neisseria gonorrhoeae*	2
*Pseudomonas aeruginosa*	1
*Helicobacter pylori*	1
*Salmonella* spp.	1

**Table 2:** Classification of explicit definitions of potentially inappropriate prescriptions of antibiotics in hospitalized older patients (caution: the definitions should not be applied to severe cases or patients with known drug allergies).

Area	Domain	Sub-Domain	Explicit Definition: “It Is Potentially Inappropriate To …”
Site of infection	Urinary tract	General	1. Prescribe nitrofurantoin for a urinary tract infection (apart from cystitis)
			2. Prescribe norfloxacin in a urinary tract infection (apart from cystitis)
			3. Prescribe amoxicillin–clavulanic acid for the empirical therapy of a urinary tract infection
		Urinary tract colonization	4. Prescribe antibiotics for urinary tract colonization (in the absence of urinary tract surgery, and regardless of the pathogen identified [ESBL, etc.])
		Cystitis	5. Prescribe aminoglycosides in a case of cystitis
			6. Prescribe the following antibiotics for the empirical therapy of cystitis: amoxicillin, amoxicillin–clavulanic acid, azithromycin, cefadroxil, cefuroxime, and doxycycline
			7. Prescribe a 3GC in a case of cystitis
			8. Prescribe a 4GC in a case of cystitis
			9. Prescribe fluoroquinolones for the first-line treatment of cystitis
		Urinary tract infections in men	10. Prescribe amoxicillin for urinary tract infections in men (apart from *Enterococci*)
			11. Prescribe amoxicillin–clavulanic acid for urinary tract infections in men
	Lower respiratory tract	Bronchitis	12. Prescribe antibiotics for acute bronchitis
		AECOPD	13. Prescribe antibiotics in the prophylaxis of AECOPD
		Pneumonia	14. Prescribe antibiotics in a case of viral pneumonia or pleurisy
			15. Prescribe amoxicillin–clavulanic acid for documented acute community-acquired pneumococcal pneumonia
			16. Prescribe ceftriaxone for documented acute community-acquired pneumococcal pneumonia
			17. Prescribe an injectable 3GC for community-acquired pneumonia without comorbidities
			18. Prescribe a 3GC–fluoroquinolone combination for the empirical therapy of pneumonia
			19. Prescribe fluoroquinolones for the first-line treatment of pneumonia
			20. Prescribe a macrolide for community-acquired pneumonia (apart from legionellosis)
			21. Prescribe antibiotics for an infiltrate on a chest X-ray in the absence of clinically significant symptoms of pneumonia
	Upper respiratory tract	Non-specific URTI	22. Prescribe antibiotics for nasopharyngitis (a common cold), acute laryngitis, and tracheitis
			23. Prescribe doxycycline in acute pharyngitis
			24. Prescribe a 3GC for an URTI
			25. Prescribe a fluoroquinolone for the first-line treatment of an URTI
		Sinusitis	26. Prescribe antibiotics in a case of acute sinusitis, with symptoms for less than 5 days and/or no fever
			27. Prescribe doxycycline in acute sinusitis
		Tonsillitis	28. Prescribe other treatments than amoxicillin and/or penicillin V for acute pharyngotonsillitis
			29. Prescribe doxycycline for acute tonsillitis
			30. Prescribe antibiotics for viral tonsillitis
		Otitis	31. Prescribe erythromycin as an empirical therapy in acute otitis media
			32. Prescribe trimethoprim/sulfamethoxazole as an empirical therapy in acute otitis media
			33. Prescribe antibiotics for uncomplicated acute otitis externa unless there is extension beyond the ear canal or the presence of specific host factors that indicate a need for systemic treatment
	Skin and soft tissues		34. Prescribe any molecule other than amoxicillin for non-necrotizing cellulitis of the lower limb (uncomplicated erysipelas)
			35. Prescribe fluoroquinolones for skin and soft tissue infections
			36. Prescribe an anti-MRSA antibiotic for community-acquired non-necrotizing cellulitis
			37. Prescribe an antibiotic for the treatment of a wound in the absence of cellulitis (apart from a bite)
			38. Prescribe topical antibiotics (apart from *Staphylococcus aureus* decontamination)
			39. Prescribe antibiotics for a decubitus ulcer in an individual at the end of life
			40. Prescribe ceftriaxone for the empirical therapy of skin and soft tissue infections in immunocompetent hosts
	Gastrointestinal tract		41. Prescribe antibiotics for the empirical therapy of diarrhea
			42. Prescribe a fluoroquinolone for the empirical therapy of a gastro-intestinal infection
			43. Prescribe amoxicillin–clavulanic acid for nosocomial gastrointestinal infections
			44. Prescribe antibiotics for the empirical therapy of acute vomiting or diarrhea in the absence of a positive stool culture or a positive toxin assay for *Clostridioides difficile*
			45. Prescribe antibiotics that do not cover the following bacteria for secondary intra-abdominal infections: aerobic, anaerobic, or beta-lactamase-producing Gram-negative bacilli
	Bones/joints		46. Prescribe ceftriaxone for the empirical therapy of a bone or joint infection in immunocompetent hosts
			47. Prescribe fluoroquinolones for the empirical therapy of a bone or joint infection
			48. Prescribe rifampicin for the empirical therapy of a bone or joint infection
			49. Prescribe antibiotics for the empirical treatment of a bone or joint infection before reliable microbiological samples have been collected
	Bloodstream		50. Initiate antibiotic therapy more than 24 h after a positive blood culture (unless the sample is contaminated)
	Dental care		51. Prescribe antibiotics for the first-line therapy of pulpitis
			52. Prescribe antibiotics for acute dental pain unless patient has facial swelling, adenopathy, difficulty opening the mouth, fever, difficulty swallowing or ulcerative gingivitis
General principles of antibiotic use	All sites of infection		53. Prescribe nitrofurantoin in men
			54. Prescribe ertapenem as a first-line treatment
			55. Prescribe aminoglycosides when the severity criteria are not met
			56. Prescribe fluoroquinolones as a first-line treatment (apart from urinary tract infections in men or acute pyelonephritis)
			57. Prescribe antibiotics for an isolated elevation of CRP
			58. Prescribe oral 3GCs (except for in a documented case of acute pyelonephritis in a woman)
			59. Prescribe fluoroquinolones for empirical treatment in patients treated with fluoroquinolones in the previous 6 months
	Undocumented infections		60. Prescribe carbapenems as an empirical therapy
			61. Prescribe ertapenem as an empirical therapy
			62. Prescribe fluoroquinolones as an empirical therapy
			63. Prescribe rifampicin as an empirical therapy
			64. Prescribe cotrimoxazole as an empirical therapy (except when pneumocystosis is suspected)
	Community-acquired infections		65. Prescribe antibiotics that are effective against methicillin-resistant *Staphylococci* (vancomycin, teicoplanin, daptomycin, linezolide, and dalbavancin) as an empirical therapy for community-acquired infections
			66. Prescribe carbapenems for a community-acquired infection
			67. Prescribe piperacillin–tazobactam for a community-acquired infection
			68. Prescribe a 4GC for a community-acquired infection
Use	Dosing		69. Reduce the dose level of aminoglycosides in the event of kidney failure
			70. Underdose gentamicin (at least 10% below the recommended dose)
			71. Fail to re-evaluate the dose level as a function of changes in renal function changes
			72. Prescribe a continuous infusion of vancomycin in the absence of a loading dose
	Duration of treatment		73. Prescribe antibiotics for more than 5 days for AECOPD
			74. Prescribe antibiotics for more than 7 days for pneumonia
			75. Prescribe antibiotics for more than 7 days for non-necrotizing cellulitis
			76. Prescribe aminoglycosides for more than 3 days
	Combination of antibiotics		77. Combine amoxicillin–clavulanic acid with fluoroquinolones
			78. Combine amoxicillin–clavulanic acid with metronidazole
			79. Combine two aminoglycosides
			80. Prescribe systemic rifampicin alone (i.e., as a monotherapy)
	Laboratory assays		81. Prescribe a glycopeptide without assaying plasma concentrations (apart from orally administered glycopeptides)
	Administration route		82. Prescribe oral penicillin M
			83. Prescribe subcutaneous ceftriaxone if intravenous administration is possible
			84. Prescribe intravenous (i.v.) antibiotics when the patient meets the criteria for use of the per os (p.o.) formulation according to the i.v.–p.o. antibiotic switch protocol: - Oral administration not compromised; - No sepsis or a deteriorating clinical condition; - No special indications (meningitis, endocarditis, immunosuppression, bone/joint infection, deep abscess); - An oral formulation of the drug is available
			85. Intravenous (i.v.) fluoroquinolones after 48 h when the patient meets the criteria for use of the per os (p.o.) formulation: - The need to continue antibiotic treatment; - Patient clinically stable; - Patient capable of tolerating the p.o. formulation; - The absence of factors that would adversely affect p.o. bioavailability (e.g., gastrointestinal abnormalities or drug interactions)
			86. Prescribe a subcutaneously administered aminoglycoside
Organisms	Viruses		87. Prescribe antibiotics for a SARS-CoV-2 infection
			88. Prescribe antibiotics for probable viral infections
			89. Prescribe antibiotics for influenza
	*Clostridioides difficile*		90. Prescribe metronidazole for a CDI
			91. Prescribe intravenous vancomycin for the treatment of a CDI
			92. Prescribe metronidazole rather than vancomycin for a severe CDI
			93. Prescribe antibiotics for empirical therapy for a mild-to-moderate CDI (i.e., not meeting the criteria for severe CDI), unless the recurrence of a recent CDI is suspected
	*Pseudomonas aeruginosa*		94. Prescribe a fluoroquinolone alone for the first-line treatment of *Pseudomonas aeruginosa* infections
	*Helicobacter pylori*		95. Prescribe an amoxicillin–clavulanic acid/tetracycline combination for the eradication of *Helicobacter pylori*
	*Salmonella*		96. Prescribe fluoroquinolones for the first-line treatment of salmonellosis
	*Neisseria gonorrhoeae*		97. Prescribe ciprofloxacin for uncomplicated gonococcal urethritis in men
			98. Prescribe amoxicillin for uncomplicated gonococcal urethritis in men
	*Treponema pallidum*		99. Prescribe ciprofloxacin for late syphilis
			100. Prescribe azithromycin for late syphilis

ESBL: extended spectrum beta-lactamase; 3GC: third-generation cephalosporin; 4GC: fourth-generation cephalosporin; URTI: upper respiratory tract infection. AECOPD: acute exacerbation of chronic obstructive pulmonary disease. MRSA: methicillin-resistant *Staphylococcus aureus*; SARS-CoV-2: severe acute respiratory syndrome coronavirus 2; CDI: *Clostridioides difficile* infection.

## Data Availability

Study data are available on request.

## Supplementary Materials

The following supporting information can be downloaded at: https://www.mdpi.com/article/10.3390/antibiotics13030283/s1, Supplementary Data S1: Preparation of the preliminary list; Supplementary Data S2: Eligible list of definitions; Supplementary Data S3: Geographical distribution of participants; Supplementary Data S4: Characteristics of participants.

## References

[1] Antimicrobial Resistance Collaborators (2022). Global burden of bacterial antimicrobial resistance in 2019: A systematic analysis. Lancet.

[2] World Health Organization (2017). Global Antimicrobial Resistance Surveillance System (GLASS) Report: Early Implementation 2016–2017.

[3] European Centre for Disease Prevention and Control, World Health Organization (2022). Antimicrobial Resistance Surveillance in Europe: 2022: 2020 Data.

[4] Willemsen I., Groenhuijzen A., Bogaers D., Stuurman A., van Keulen P., Kluytmans J. (2007). Appropriateness of Antimicrobial Therapy Measured by Repeated Prevalence Surveys. Antimicrob. Agents Chemother..

[5] World Health Organization (2021). WHO Strategic Priorities on Antimicrobial Resistance: Preserving Antimicrobials for Today and Tomorrow.

[6] European Centre for Disease Prevention and Control, European Medicines Agency (2009). The Bacterial Challenge: Time to React: A Call to Narrow the Gap between Multidrug-Resistant Bacteria in the EU and the Development of New Antibacterial Agents.

[7] (2021). Antimicrobial Stewardship Interventions: A Practical Guide.

[8] American Geriatrics Society Beers Criteria^®^ Update Expert Panel (2019). American Geriatrics Society 2019 Updated AGS Beers Criteria^®^ for Potentially Inappropriate Medication Use in Older Adults. J. Am. Geriatr. Soc..

[9] Laroche M.-L., Charmes J.-P., Merle L. (2007). Potentially inappropriate medications in the elderly: A French consensus panel list. Eur. J. Clin. Pharmacol..

[10] O’Mahony D., O’Sullivan D., Byrne S., O’Connor M.N., Ryan C., Gallagher P. (2015). STOPP/START criteria for potentially inappropriate prescribing in older people: Version 2. Age Ageing.

[11] Guaraldo L., Cano F.G., Damasceno G.S., Rozenfeld S. (2011). Inappropriate medication use among the elderly: A systematic review of administrative databases. BMC Geriatr..

[12] Beuscart J.-B., Genin M., Dupont C., Verloop D., Duhamel A., Defebvre M.-M., Puisieux F. (2017). Potentially inappropriate medication prescribing is associated with socioeconomic factors: A spatial analysis in the French Nord-Pas-de-Calais Region. Age Ageing.

[13] Mor A., Frøslev T., Thomsen R.W., Oteri A., Rijnbeek P., Schink T., Garbe E., Pecchioli S., Innocenti F., Bezemer I. (2015). Antibiotic use varies substantially among adults: A cross-national study from five European Countries in the ARITMO project. Infection.

[14] Scott M.M., Liang S.Y. (2021). Infections in Older Adults. Emerg. Med. Clin. N. Am..

[15] Cavalié P., Hider-Mlynarz K. (2017). L’évolution des Consommations D’antibiotiques en France Entre 2000 et 2015.

[16] Gavazzi G., Krause K.-H. (2002). Ageing and infection. Lancet Infect. Dis..

[17] Baclet N., Ficheur G., Alfandari S., Ferret L., Senneville E., Chazard E., Beuscart J.-B. (2017). Explicit definitions of potentially inappropriate prescriptions of antibiotics in older patients: A compilation derived from a systematic review. Int. J. Antimicrob. Agents.

[18] Motter F.R., Fritzen J.S., Hilmer S.N., Paniz É.V., Paniz V.M.V. (2018). Potentially inappropriate medication in the elderly: A systematic review of validated explicit criteria. Eur. J. Clin. Pharmacol..

[19] Dalkey N.C. The Delphi Method. https://www.rand.org/pubs/research_memoranda/RM5888.html.

[20] Diamond I.R., Grant R.C., Feldman B.M., Pencharz P.B., Ling S.C., Moore A.M., Wales P.W. (2014). Defining consensus: A systematic review recommends methodologic criteria for reporting of Delphi studies. J. Clin. Epidemiol..

[21] Slade S.C., Dionne C.E., Underwood M., Buchbinder R. (2014). Standardised method for reporting exercise programmes: Protocol for a modified Delphi study. BMJ Open.

[22] Boulkedid R., Abdoul H., Loustau M., Sibony O., Alberti C. (2011). Using and Reporting the Delphi Method for Selecting Healthcare Quality Indicators: A Systematic Review. PLoS ONE.

[23] Sinha I.P., Smyth R.L., Williamson P.R. (2011). Using the Delphi Technique to Determine Which Outcomes to Measure in Clinical Trials: Recommendations for the Future Based on a Systematic Review of Existing Studies. PLoS Med..

[24] Brook R.H. (1977). Quality—Can We Measure It?. N. Engl. J. Med..

[25] Spinewine A., Schmader K.E., Barber N., Hughes C., Lapane K.L., Swine C., Hanlon J.T. (2007). Appropriate prescribing in elderly people: How well can it be measured and optimised?. Lancet.

[26] Baclet N., Calafiore M., Fregnac C., Gavazzi G., Forestier E., Roubaud-Baudron C., Fraisse T., Alfandari S., Senneville E., Beuscart J.-B. (2022). Explicit definitions of potentially inappropriate prescriptions of antibiotics in hospitalized older patients. Infect. Dis. Now.

[27] Géodes—Santé Publique France—Indicateurs: Cartes, Données et Graphiques. https://geodes.santepubliquefrance.fr/#c=home.

[28] Likert R. (1932). A Technique for the Measurement of Attitudes.

[29] R Core Team (2013). R: A Language and Environment for Statistical Computing.

[30] Earl T.R., Katapodis N.D., Schneiderman S.R., Shoemaker-Hunt S.J. (2020). Using Deprescribing Practices and the Screening Tool of Older Persons’ Potentially Inappropriate Prescriptions Criteria to Reduce Harm and Preventable Adverse Drug Events in Older Adults. J. Patient Saf..

[31] Dalton K., O’Brien G., O’Mahony D., Byrne S. (2018). Computerised interventions designed to reduce potentially inappropriate prescribing in hospitalised older adults: A systematic review and meta-analysis. Age Ageing.

[32] Hill-Taylor B., Walsh K.A., Stewart S., Hayden J., Byrne S., Sketris I.S. (2016). Effectiveness of the STOPP/START (Screening Tool of Older Persons’ potentially inappropriate Prescriptions/Screening Tool to Alert doctors to the Right Treatment) criteria: Systematic review and meta-analysis of randomized controlled studies. J. Clin. Pharm. Ther..

[33] World Health Organization (2019). Critically Important Antimicrobials for Human Medicine.

[34] Gauzit R., Castan B., Bonnet E., Bru J.P., Cohen R., Diamantis S., Faye A., Hitoto H., Issa N., Lebeaux D. (2021). Anti-infectious treatment duration: The SPILF and GPIP French guidelines and recommendations. Infect. Dis. Now.

[35] Wintenberger C., Guery B., Bonnet E., Castan B., Cohen R., Diamantis S., Lesprit P., Maulin L., Péan Y., Peju E. (2017). Proposal for shorter antibiotic therapies. Médecine Mal. Infect..

[36] Hanretty A.M., Gallagher J.C. (2018). Shortened Courses of Antibiotics for Bacterial Infections: A Systematic Review of Randomized Controlled Trials. Pharmacother. J. Hum. Pharmacol. Drug Ther..

[37] Mendelson M., Morris A.M., Thursky K., Pulcini C. (2020). How to start an antimicrobial stewardship programme in a hospital. Clin. Microbiol. Infect..

[38] CDC (2019). The Core Elements of Hospital Antibiotic Stewardship Programs.

[39] Scott I.A., Pillans P.I., Barras M., Morris C. (2018). Using EMR-enabled computerized decision support systems to reduce prescribing of potentially inappropriate medications: A narrative review. Ther. Adv. Drug Saf..

[40] Cuvelier E., Robert L., Musy E., Rousselière C., Marcilly R., Gautier S., Odou P., Beuscart J.-B., Décaudin B. (2021). The clinical pharmacist’s role in enhancing the relevance of a clinical decision support system. Int. J. Med. Inf..

[41] Robert L., Quindroit P., Henry H., Perez M., Rousselière C., Lemaitre M., Vambergue A., Décaudin B., Beuscart J.-B. (2024). Implementation of a clinical decision support system for the optimization of antidiabetic drug orders by pharmacists. Br. J. Clin. Pharmacol..

[42] Robert L., Cuvelier E., Rousselière C., Gautier S., Odou P., Beuscart J.-B., Décaudin B. (2023). Detection of Drug-Related Problems through a Clinical Decision Support System Used by a Clinical Pharmacy Team. Healthcare.

