# Supplementary material for: One Hundred Explicit Definitions of Potentially Inappropriate Prescriptions of Antibiotics in Hospitalized Older Patients: The Results of an Expert Consensus Study
Source: Antibiotics (Basel). 2024 Mar 20;13(3):283. doi: 10.3390/antibiotics13030283 (PMC10967330; doi:10.3390/antibiotics13030283)
Supplement: Supplementary file 1 [file antibiotics-13-00283-s001.zip › SuppData1_PreparationOfPreliminaryList.docx]

One Hundred Explicit Definitions of Potentially Inappropriate Prescriptions of Antibiotics in Hospitalized Older Patients: The Results of an Expert Consensus Study

Nicolas Baclet ^1,2,^*, Emmanuel Forestier ^3^, Gaëtan Gavazzi ^4^, Claire Roubaud-Baudron ^5^, Vincent Hiernard ^1^,
Rozenn Hequette-Ruz ^6^, Serge Alfandari ^7^, Hugues Aumaître ^8^, Elisabeth Botelho-Nevers ^9,10,11^, Pauline Caraux-Paz ^12^, Alexandre Charmillon ^13,14^, Sylvain Diamantis ^15,16^, Thibaut Fraisse ^17^, Pierre Gazeau ^18^, Maxime Hentzien ^19,20^,
Jean-Philippe Lanoix ^21,22^, Marc Paccalin ^23,24^, Alain Putot ^25,26^, Yvon Ruch ^27^, Eric Senneville ^7^
and Jean-Baptiste Beuscart ^1^ on behalf of the GInGer (SPILF–SFGG Study Group) ^†^

^1^ CHU Lille, University of Lille, F-59000 Lille, France; [baclet.nicolas@ghicl.net](mailto:baclet.nicolas@ghicl.net) (N.B.); hiernard-v@ch-valenciennes.fr (V.H.);
jean-baptiste.beuscart@univ-lille.fr (J.-B.B.)

^2^ Groupe Hospitalier de l’Institut Catholique (GHICL), Service de Maladies Infectieuses, Université
Catholique de Lille, F-59160 Lille, France

^3^ Service de Maladies Infectieuses, Centre Hospitalier Métropole Savoie, F-73000 Chambéry, France;
emmanuel.forestier@ch-metropole-savoie.fr

^4^ Clinique Universitaire de Médecine Gériatrique, Centre Hospitalier Universitaire de Grenoble-Alpes,
GREPI EA7408 Université Grenoble-Alpes, F-38000 Grenoble, France; ggavazzi@chu-grenoble.fr

^5^ CHU Bordeaux, Pôle de Gérontologie Clinique, University of Bordeaux, INSERM 1312 BRIC,
F-33000 Bordeaux, France; claire.roubaud@chu-bordeaux.fr

^6^ Service de Maladies Infectieuses, CH Roubaix, F-59056 Roubaix, France; rozenn.hequette@ch-roubaix.fr

^7^ Service Universitaire de Maladies Infectieuses et Tropicales, Hôpital Gustave Dron, F-59200 Tourcoing, France; salfandari@ch-tourcoing.fr (S.A.); esenneville@ch-tourcoing.fr (E.S.)

^8^ Service de Maladies Infectieuses et Tropicales, Centre Hospitalier de Perpignan, F-66000 Perpignan, France; hugues.aumaitre@ch-perpignan.fr

^9^ Infectious Diseases Department, University Hospital of Saint-Etienne, GIMAP (EA 3064), F-42055 Cedex 02 Saint-Etienne, , France; elisabeth.botelho-nevers@chu-st-etienne.fr

^10^ University of Saint-Etienne, Faculty of Medicine of Saint-Etienne, F-42023 Cedex 02 Saint-Etienne, France

^11^ University of Lyon, F-69000 Lyon, France

^12^ Service de Maladies Infectieuses et Tropicales, Hôpital Intercommunal de Villeneuve-Saint-Georges,
F-94190 Villeneuve-Saint-Georges, France; pauline.caraux-paz@chiv.fr

^13^ CHRU-Nancy, Infectious Diseases Department, F-54000 Nancy, France; a.charmillon@chru-nancy.fr

^14^ Grand Est Antibiotic Stewardship Network Coordinator, AntibioEst, F-54000 Nancy, France

^15^ Service de Maladies Infectieuses et Tropicales, Hôpital de Melun, F-77000 Melun, France;
sylvain.diamantis@ghsif.fr

^16^ Unité de Recherche DYNAMIC, Université Paris-Est Créteil, F-94000 Créteil, France

^17^ Court Séjour Gériatrique Aigu, Centre Hospitalier Alès-Cévennes, F-30100 Alès, France; tfraisse@yahoo.fr

^18^ Service des Maladies Infectieuses et Tropicales, CHRU de Brest, F-29609 Brest Cedex, France; pierre.gazeau@chu-brest.fr

^19^ Department of Internal Medicine, Infectious Diseases and Clinical Immunology, University Hospital of Reims, F-51100 Reims, France; mhentzien@chu-reims.fr

^20^ EA3797-Viellissement Fragilité, Reims Champagne Ardennes University, F-51100 Reims, France

^21^ AGIR UR 4294, University Picardie Jules Verne, F-80000 Amiens, France; lanoix.jean-philippe@chu-amiens.fr

^22^ Department of Infectious Diseases, Amiens University Hospital, F-80000 Amiens, France

^23^ Pôle de Gériatrie, CHU Poitiers, Université Poitiers, F-86000 Poitiers, France

^24^ Centre d’Investigation Clinique CIC 1402, INSERM CHU Poitiers, Université Poitiers, F-86000 Poitiers, France; marc.paccalin@chu-poitiers.fr

^25^ Médecine Interne et Maladies Infectieuses, Hôpitaux du Pays du Mont Blanc, F-74700 Sallanches, France

^26^ Physiopathologie et Epidémiologie Cérébro-Cardiovasculaires, Université de Bourgogne, F-21000 Dijon, France;
aputot@ch-sallanches-chamonix.fr

^27^ Department of Infectious Diseases, Strasbourg University Hospital, F-67000 Strasbourg, France;
yvon.ruch@chru-strasbourg.fr

***** Correspondence: baclet.nicolas@ghicl.net; Tel.: +33-320-626-969; Fax: +33-320-626-881

^†^ Membership of the GInGer is provided in the Acknowledgments.

Supplementary Data S1

Preparation of the list of eligible explicit definitions for the Delphi survey.

The principle underlying our Delphi survey was the collection of the opinions of a panel of experts regarding their agreement or disagreement with a list of eligible explicit definitions of potentially inappropriate prescriptions of antibiotics (antibiotic-PIPs) for hospitalized older patients. As in most Delphi surveys, only explicit definitions that achieved a consensus were selected. The steps through which the list of eligible explicit definitions was prepared prior to submission to the participants in the Delphi survey are detailed below and shown in Figure SD1.

1. Sources of explicit definitions included in the Delphi survey (Figure SD1, Step 1).

The list of explicit definitions of antibiotic-PIPs suggested to the expert panel was prepared in two preliminary studies. Firstly, a systematic review of the literature identified a list of 62 explicit definitions of antibiotic-PIPs [17]. Secondly, a complementary qualitative study in France (conducted with infectious diseases experts and geriatricians), led to the development of a list of 65 explicit definitions of antibiotic-PIPs specifically for hospitalized older patients [26].

2. Preparation of a list of explicit definitions submitted to the participants in the Delphi survey (Figure SD1, Step 2).

The explicit definitions of antibiotic-PIPs from the two preliminary studies were used to draw up a list for submission to the participants in the Delphi survey. Two researchers (a French-speaking infectious disease physician and an English-speaking infectious disease physician) prepared the list of definitions independently, in the following steps:

- The definitions from the systematic review of the English literature were translated into French.

During this stage, the two researchers checked that the French translation was representative of the English wording associated with each definition. It is noteworthy that during the systematic review of the literature, the wording of the definitions had been validated by the investigators and a steering committee [17].

- After translation into French, the list from the systematic review of the literature was compared with the list from the qualitative study independently by two researchers (NB and RHR).

Each of the two researchers assessed the two lists, in order to (i) identify and group together similar definitions in the two lists (a new formulation was developed so as to include all the notions and elements contained in each of the source definitions) and (ii) identify and then reject explicit definitions that were outside the scope of the study. Differences of opinion between the two researchers were discussed, resolved by consensus, and then validated by the steering committee.

3. Validation of the list of eligible explicit definitions of antibiotic-PIPs (Figure SD1, Step 3)

In order to validate the preliminary list of explicit definitions, we contacted a regional group of infectious disease experts and geriatricians. Two infectious disease specialists and two geriatricians formed two infectious disease specialist–geriatrician pairings. Each pair reviewed each definition in two stages. During the first stage, the experts in each pair reviewed the definitions independently. Differences of opinion were resolved by discussion and consensus. In the second step, the pairs compared their respective reviews of each definition. Differences of opinion were again resolved by discussion and consensus at a joint meeting of the four participants, which was facilitated by two researchers (NB and RHR). Any remaining differences of opinion were resolved by the steering committee.

Validation

Preparation of the list

Preliminary studies

Systematic review of the literature

Qualitative study

65 explicit definitions of antibiotic-PIPs (French)

62 explicit definitions of antibiotic-PIPs (English)

**Preliminary list of 103 explicit definitions of antibiotic-PIPs**

Translation into French

Preliminary list of 103 explicit definitions

Exclusion of 15 definitions

(outside the scope of the study)

Researcher 1

Researcher 2

Researcher 1

Researcher 2

Grouping of the 127 explicit definitions of antibiotic-PIPs

Independent expert group

$$✔$$

steering committee

$$✔$$

steering committee

Merger of 9 definitions from each list

**Figure SD1.** Flow chart of the preparation of the list of eligible explicit definitions of potentially inappropriate antibiotic prescriptions (antibiotic-PIPs) for the Delphi survey.
