# Supplementary material for: One Hundred Explicit Definitions of Potentially Inappropriate Prescriptions of Antibiotics in Hospitalized Older Patients: The Results of an Expert Consensus Study
Source: Antibiotics (Basel). 2024 Mar 20;13(3):283. doi: 10.3390/antibiotics13030283 (PMC10967330; doi:10.3390/antibiotics13030283)
Supplement: Supplementary file 1 [file antibiotics-13-00283-s001.zip › SuppData2_EligibleListOfDefinitions.docx]

One Hundred Explicit Definitions of Potentially Inappropriate Prescriptions of Antibiotics in Hospitalized Older Patients: The Results of an Expert Consensus Study

Nicolas Baclet ^1,2,^*, Emmanuel Forestier ^3^, Gaëtan Gavazzi ^4^, Claire Roubaud-Baudron ^5^, Vincent Hiernard ^1^,
Rozenn Hequette-Ruz ^6^, Serge Alfandari ^7^, Hugues Aumaître ^8^, Elisabeth Botelho-Nevers ^9,10,11^, Pauline Caraux-Paz ^12^, Alexandre Charmillon ^13,14^, Sylvain Diamantis ^15,16^, Thibaut Fraisse ^17^, Pierre Gazeau ^18^, Maxime Hentzien ^19,20^,
Jean-Philippe Lanoix ^21,22^, Marc Paccalin ^23,24^, Alain Putot ^25,26^, Yvon Ruch ^27^, Eric Senneville ^7^
and Jean-Baptiste Beuscart ^1^ on behalf of the GInGer (SPILF–SFGG Study Group) ^†^

^1^ CHU Lille, University of Lille, F-59000 Lille, France; [baclet.nicolas@ghicl.net](mailto:baclet.nicolas@ghicl.net) (N.B.); hiernard-v@ch-valenciennes.fr (V.H.);
jean-baptiste.beuscart@univ-lille.fr (J.-B.B.)

^2^ Groupe Hospitalier de l’Institut Catholique (GHICL), Service de Maladies Infectieuses, Université
Catholique de Lille, F-59160 Lille, France

^3^ Service de Maladies Infectieuses, Centre Hospitalier Métropole Savoie, F-73000 Chambéry, France;
emmanuel.forestier@ch-metropole-savoie.fr

^4^ Clinique Universitaire de Médecine Gériatrique, Centre Hospitalier Universitaire de Grenoble-Alpes,
GREPI EA7408 Université Grenoble-Alpes, F-38000 Grenoble, France; ggavazzi@chu-grenoble.fr

^5^ CHU Bordeaux, Pôle de Gérontologie Clinique, University of Bordeaux, INSERM 1312 BRIC,
F-33000 Bordeaux, France; claire.roubaud@chu-bordeaux.fr

^6^ Service de Maladies Infectieuses, CH Roubaix, F-59056 Roubaix, France; rozenn.hequette@ch-roubaix.fr

^7^ Service Universitaire de Maladies Infectieuses et Tropicales, Hôpital Gustave Dron, F-59200 Tourcoing, France; salfandari@ch-tourcoing.fr (S.A.); esenneville@ch-tourcoing.fr (E.S.)

^8^ Service de Maladies Infectieuses et Tropicales, Centre Hospitalier de Perpignan, F-66000 Perpignan, France; hugues.aumaitre@ch-perpignan.fr

^9^ Infectious Diseases Department, University Hospital of Saint-Etienne, GIMAP (EA 3064), F-42055 Cedex 02 Saint-Etienne, , France; elisabeth.botelho-nevers@chu-st-etienne.fr

^10^ University of Saint-Etienne, Faculty of Medicine of Saint-Etienne, F-42023 Cedex 02 Saint-Etienne, France

^11^ University of Lyon, F-69000 Lyon, France

^12^ Service de Maladies Infectieuses et Tropicales, Hôpital Intercommunal de Villeneuve-Saint-Georges,
F-94190 Villeneuve-Saint-Georges, France; pauline.caraux-paz@chiv.fr

^13^ CHRU-Nancy, Infectious Diseases Department, F-54000 Nancy, France; a.charmillon@chru-nancy.fr

^14^ Grand Est Antibiotic Stewardship Network Coordinator, AntibioEst, F-54000 Nancy, France

^15^ Service de Maladies Infectieuses et Tropicales, Hôpital de Melun, F-77000 Melun, France;
sylvain.diamantis@ghsif.fr

^16^ Unité de Recherche DYNAMIC, Université Paris-Est Créteil, F-94000 Créteil, France

^17^ Court Séjour Gériatrique Aigu, Centre Hospitalier Alès-Cévennes, F-30100 Alès, France; tfraisse@yahoo.fr

^18^ Service des Maladies Infectieuses et Tropicales, CHRU de Brest, F-29609 Brest Cedex, France; pierre.gazeau@chu-brest.fr

^19^ Department of Internal Medicine, Infectious Diseases and Clinical Immunology, University Hospital of Reims, F-51100 Reims, France; mhentzien@chu-reims.fr

^20^ EA3797-Viellissement Fragilité, Reims Champagne Ardennes University, F-51100 Reims, France

^21^ AGIR UR 4294, University Picardie Jules Verne, F-80000 Amiens, France; lanoix.jean-philippe@chu-amiens.fr

^22^ Department of Infectious Diseases, Amiens University Hospital, F-80000 Amiens, France

^23^ Pôle de Gériatrie, CHU Poitiers, Université Poitiers, F-86000 Poitiers, France

^24^ Centre d’Investigation Clinique CIC 1402, INSERM CHU Poitiers, Université Poitiers, F-86000 Poitiers, France; marc.paccalin@chu-poitiers.fr

^25^ Médecine Interne et Maladies Infectieuses, Hôpitaux du Pays du Mont Blanc, F-74700 Sallanches, France

^26^ Physiopathologie et Epidémiologie Cérébro-Cardiovasculaires, Université de Bourgogne, F-21000 Dijon, France;
aputot@ch-sallanches-chamonix.fr

^27^ Department of Infectious Diseases, Strasbourg University Hospital, F-67000 Strasbourg, France;
yvon.ruch@chru-strasbourg.fr

***** Correspondence: baclet.nicolas@ghicl.net; Tel.: +33-320-626-969; Fax: +33-320-626-881

^†^ Membership of the GInGer is provided in the Acknowledgments.

**Supplementary Data S2.** List of eligible explicit definitions of potentially inappropriate antibiotic prescriptions (antibiotic-PIPs) for the Delphi survey.

| Area | Domain | Sub-domain | Explicit definition :  "It is potentially inappropriate to …" |
| --- | --- | --- | --- |
| Site of infection | Urinary tract | General | 1. prescribe nitrofurantoin for a urinary tract infection (apart from cystitis) |
|  |  |  | 2. prescribe norfloxacin in a urinary tract infection (apart from cystitis) |
|  |  |  | 3. prescribe amoxicillin-clavulanic acid for the empiric therapy of a urinary tract infection |
|  |  |  | 4. prescribe fluoroquinolones for the empirical therapy of urinary tract infections |
|  |  | Urinary tract colonization | 5. prescribe antibiotics for urinary tract colonization (in the absence of urinary tract surgery, and regardless of the pathogen identified [ESBL, etc.]) |
|  |  | Cystitis | 6. prescribe a 3GC in a case of cystitis |
|  |  |  | 7. prescribe a 4GC in a case of cystitis |
|  |  |  | 8. prescribe fluoroquinolones for the first-line treatment of cystitis |
|  |  |  | 9. prescribe the following antibiotics for the empiric therapy of cystitis: amoxicillin, amoxicillin/clavulanic acid, fosfomycin, azithromycin, cefadroxil, cefuroxime, and doxycycline |
|  |  | Urinary tract infections in men | 10. prescribe amoxicillin for urinary tract infections in men (apart from *Enterococci*) |
|  |  |  | 11. prescribe amoxicillin-clavulanic acid for urinary tract infections in men |
|  | Lower respiratory tract | Bronchitis | 12. prescribe antibiotics for bronchitis |
|  |  | AECOPD | 13. prescribe antibiotics in the prophylaxis of AECOPD |
|  |  | Pneumonia | 14. prescribe ceftriaxone for documented acute community-acquired pneumococcal pneumonia |
|  |  |  | 15. prescribe amoxicillin-clavulanic acid for documented acute community-acquired pneumococcal pneumonia |
|  |  |  | 16. prescribe an injectable 3GC for community-acquired pneumonia without comorbidities |
|  |  |  | 17. prescribe fluoroquinolones for the first-line treatment of community-acquired pneumonia |
|  |  |  | 18. prescribe a macrolide for community-acquired pneumonia (apart from legionellosis) |
|  |  |  | 19. prescribe a two-antibiotic combination in a case of pneumonia |
|  |  |  | 20. prescribe antibiotics in a case of viral pneumonia or pleurisy |
|  |  |  | 21. prescribe antibiotics for an infiltrate on a chest X-ray in the absence of clinically significant symptoms of pneumonia |
|  |  |  | 22. prescribe antibiotics for respiratory symptoms in advanced dementia at the end of life |
|  |  |  | 23. prescribe the following single antibiotics for community-acquired pneumonia: cefaclor, ceftriaxone, cloxacillin, minocycline, tetracycline, vancomycin, sulfamethoxazole, sulfasalazine, trimethoprim, nitrofurantoin, ciprofloxacin, norfloxacin, ofloxacin, metronidazole, gentamicin, tobramycin, and clindamycin |
|  | Upper respiratory tract | Non-specific URTI | 24. prescribe antibiotic for nasopharyngitis (a common cold), acute laryngitis and tracheitis |
|  |  |  | 25. prescribe doxycycline in acute pharyngitis |
|  |  |  | 26. prescribe a 3GC for an URTI |
|  |  |  | 27. prescribe a fluoroquinolone for the first-line treatment of an URTI |
|  |  | Tonsillitis | 28. prescribe antibiotics for viral tonsillitis |
|  |  |  | 29. prescribe doxycycline for acute tonsillitis |
|  |  |  | 30. prescribe other treatments than amoxicillin and/or penicillin V for acute pharyngotonsillitis |
|  |  | Sinusitis | 31. prescribe amoxicillin-clavulanic acid in a case of maxillary sinusitis |
|  |  |  | 32. prescribe doxycycline in acute sinusitis |
|  |  |  | 33. prescribe antibiotics in a case of sinusitis with symptoms for less than 5 days and/or no fever |
|  |  | Otitis | 34. prescribe amoxicillin-clavulanic acid in a case of acute otitis media |
|  |  |  | 35. prescribe systemic antibiotics for uncomplicated acute otitis externa unless there is extension beyond the ear canal or the presence of specific host factors that indicate a need for systemic treatment |
|  |  |  | 36. prescribe erythromycin as an empirical treatment in acute otitis media |
|  |  |  | 37. prescribe trimethoprim/sulfamethoxazole as an empirical treatment in acute otitis media |
|  | Skin and soft tissues |  | 38. prescribe an antibiotic for the treatment of a wound in the absence of cellulitis |
|  |  |  | 39. prescribe any molecule other than amoxicillin for cellulitis of the lower limb |
|  |  |  | 40. prescribe topical antibiotics (apart from *Staphylococcus aureus* decontamination) |
|  |  |  | 41. prescribe antibiotics for a localized abscess <5 cm in diameter and with no significant surrounding cellulitis |
|  |  |  | 42. prescribe antibiotics for a decubitus ulcer in an individual at the end of life |
|  |  |  | 43. prescribe ceftriaxone for the empirical treatment of skin and soft tissue infections in immunocompetent hosts |
|  | Gastrointestinal tract |  | 44. prescribe routine empirical use of antibiotics for infectious diarrhoea |
|  |  |  | 45. prescribe amoxicillin-clavulanic acid for nosocomial gastrointestinal infections |
|  |  |  | 46. prescribe amoxicillin-clavulanic acid for the first-line treatment of digestive tract infections |
|  |  |  | 47. prescribe antibiotics for acute vomiting or diarrhoea in the absence of a positive culture for *Shigella* or *Salmonella* or a positive toxin assay for *Clostridioides difficile* |
|  |  |  | 48. prescribe antibiotics that do not cover the following bacteria for secondary intra-abdominal infections: β-lactamase-producing Gram-negative bacilli, and anaerobic micro-organisms |
|  | Bones/joints |  | 49. prescribe antibiotics for the empirical therapy of bone or joint infections before reliable microbiological samples have been collected |
|  |  |  | 50. prescribe ceftriaxone for the empirical treatment of osteomyelitis in immunocompetent hosts |
|  | Bloodstream |  | 51. prescribe antibiotics more than 24 h after blood cultures are drawn |
|  | Dental care |  | 52. prescribe antibiotics for acute dental pain unless patient has facial swelling, lymphadenopathy, difficulty opening the mouth, difficulty swallowing, fever, or acute necrotizing ulcerative gingivitis |
|  |  |  | 53. prescribe antibiotics for the first-line treatment of pulpitis |
| General principles of antibiotic use | All sites of infection |  | 54. prescribe ceftriaxone rather than cefotaxime when venous access is available |
|  |  |  | 55. prescribe oral 3GCs (except for a documented switch in a case of acute pyelonephritis in a woman) |
|  |  |  | 56. prescribe ertapenem as a first-line treatment |
|  |  |  | 57. prescribe aminoglycosides when the severity criteria are not met |
|  |  |  | 58. prescribe fluoroquinolones as a first-line treatment (apart from urinary tract infections in men or acute pyelonephritis) |
|  |  |  | 59. prescribe antibiotics for an isolated elevation of CRP |
|  |  |  | 60. prescribe imipenem if meropenem can be used instead |
|  |  |  | 61. prescribe fluoroquinolones as an empirical therapy in patients treated with fluoroquinolones in the previous 6 months |
|  |  |  | 62. prescribe a fluoroquinolone if a 3GC can be used instead |
|  | Undocumented infections |  | 63. prescribe rifampicin as an empirical therapy |
|  |  |  | 64. prescribe carbapenems as an empirical therapy |
|  |  |  | 65. prescribe ertapenem as an empirical therapy |
|  |  |  | 66. prescribe fluoroquinolones as an empirical therapy |
|  |  |  | 67. prescribe cotrimoxazole as an empirical therapy (except when pneumocystosis is suspected) |
|  | Community-acquired infections |  | 68. prescribe piperacillin-tazobactam for community-acquired infections |
|  |  |  | 69. prescribe a 4GC for community-acquired infections |
|  |  |  | 70. prescribe carbapenems for community-acquired infections |
|  |  |  | 71. prescribe antibiotics that are effective against methicillin-resistant staphylococci (vancomycin, teicoplanin, daptomycin, linezolid, and dalbavancin) as an empirical therapy for community-acquired infections |
| Use | Dosing |  | 72. use the Cockcroft-Gault equation to estimate renal function for antibiotic dose adjustments |
|  |  |  | 73. reduce the dose level of aminoglycosides in the event of kidney failure |
|  |  |  | 74. underdose gentamicin (≤ 90% of the advised dose) |
|  |  |  | 75. fail to re-evaluate the dose level as a function of changes in renal function changes |
|  |  |  | 76. prescribe rifampicin at a dose level of 20 mg/kg/day |
|  |  |  | 77. prescribe vancomycin without a loading dose |
|  | Duration of treatment |  | 78. prescribe aminoglycosides for more than 3 days |
|  |  |  | 79. prescribe aminoglycosides for more than a day |
|  |  |  | 80. prescribe a course of antibiotics for more than 7 days |
|  |  |  | 81. prescribe a course of antibiotics for more than 7 days for pneumonia, apart from documented *Pseudomonas aeruginosa* pneumonia |
|  | Combination of antibiotics |  | 82. combine amoxicillin-clavulanic acid with metronidazole |
|  |  |  | 83. combine two aminoglycosides |
|  |  |  | 84. prescribe rifampicin alone (i.e. as monotherapy) |
|  | Laboratory assays |  | 85. assay the peak and residual plasma concentrations of aminoglycosides |
|  |  |  | 86. prescribe a glycopeptide without assaying the plasma concentration |
|  | Administration route |  | 87. prescribe a subcutaneously administered aminoglycoside |
|  |  |  | 88. maintain intravenous (i.v.) ciprofloxacin after 48 h when the patient meets the criteria for use of the *per os* (p.o.) formulation:  - the need to continue antibiotic treatment  - clinically stable  - capable of tolerating the p.o. formulation  - the absence of factors that would adversely affect p.o. bioavailability (e.g. gastrointestinal abnormalities or drug interactions) |
|  |  |  | 89. prescribe i.v. antibiotics when the patient meets the criteria for use of the p.o. formulation, according to the i.v.–p.o. antibiotic switch protocol:  - oral administration not compromised  - no sepsis or a deteriorating clinical condition  - no special indications (meningitis, endocarditis, immunosuppression, bone/joint infection, deep abscess, and cystic fibrosis)  - an oral formulation of the drug is available |
| Organisms | Viruses |  | 90. prescribe antibiotics for probable viral infections |
|  |  |  | 91. prescribe antibiotics for influenza unless patient has a high temperature, severe cough, or an abscess |
|  | *Clostridioides difficile* |  | 92. prescribe metronidazole for a CDI |
|  |  |  | 93. prescribe metronidazole rather than vancomycin for a severe CDI |
|  |  |  | 94. prescribe antibiotics for empirical therapy of a mild-to moderate CDI (i.e. not meeting the criteria for severe CDI), unless the recurrence of a recent CDI is suspected |
|  | *Helicobacter pylori* |  | 95. prescribe an amoxicillin/clavulanate/tetracycline combination for the eradication of *H. pylori* |
|  | *Neisseria gonorrhoeae* |  | 96. prescribe ciprofloxacin for uncomplicated gonococcal urethritis in men |
|  |  |  | 97. prescribe amoxicillin for uncomplicated gonococcal urethritis in men |
|  |  |  | 98. prescribe azithromycin for uncomplicated gonococcal urethritis in men |
|  |  |  | 99. prescribe doxycycline for uncomplicated gonococcal urethritis in men |
|  | *Pseudomonas* |  | 100. prescribe a fluoroquinolone alone for the first-line treatment of a *Pseudomonas aeruginosa* infection |
|  | *Salmonella* |  | 101. prescribe fluoroquinolones for the first-line treatment of salmonellosis |
|  | *Treponema pallidum* |  | 102. prescribe azithromycin for late syphilis |
|  |  |  | 103. prescribe ciprofloxacin for late syphilis |

ES0BL: extended-spectrum beta-lactamase; 3GC: third-generation cephalosporin; 4GC: fourth-generation cephalosporin; URTI: upper respiratory tract infection. LRTI: lower respiratory tract infection; AECOPD: acute exacerbation of chronic obstructive pulmonary disease. CDI: *Clostridioides difficile* infection.
