# Supplementary material for: One Hundred Explicit Definitions of Potentially Inappropriate Prescriptions of Antibiotics in Hospitalized Older Patients: The Results of an Expert Consensus Study
Source: Antibiotics (Basel). 2024 Mar 20;13(3):283. doi: 10.3390/antibiotics13030283 (PMC10967330; doi:10.3390/antibiotics13030283)
Supplement: Supplementary file 1 [file antibiotics-13-00283-s001.zip › SuppData4_CaracteristicsOfTheParticipants.docx]

One Hundred Explicit Definitions of Potentially Inappropriate Prescriptions of Antibiotics in Hospitalized Older Patients: The Results of an Expert Consensus Study

Nicolas Baclet ^1,2,^*, Emmanuel Forestier ^3^, Gaëtan Gavazzi ^4^, Claire Roubaud-Baudron ^5^, Vincent Hiernard ^1^,
Rozenn Hequette-Ruz ^6^, Serge Alfandari ^7^, Hugues Aumaître ^8^, Elisabeth Botelho-Nevers ^9,10,11^, Pauline Caraux-Paz ^12^, Alexandre Charmillon ^13,14^, Sylvain Diamantis ^15,16^, Thibaut Fraisse ^17^, Pierre Gazeau ^18^, Maxime Hentzien ^19,20^,
Jean-Philippe Lanoix ^21,22^, Marc Paccalin ^23,24^, Alain Putot ^25,26^, Yvon Ruch ^27^, Eric Senneville ^7^
and Jean-Baptiste Beuscart ^1^ on behalf of the GInGer (SPILF–SFGG Study Group) ^†^

^1^ CHU Lille, University of Lille, F-59000 Lille, France; [baclet.nicolas@ghicl.net](mailto:baclet.nicolas@ghicl.net) (N.B.); hiernard-v@ch-valenciennes.fr (V.H.);
jean-baptiste.beuscart@univ-lille.fr (J.-B.B.)

^2^ Groupe Hospitalier de l’Institut Catholique (GHICL), Service de Maladies Infectieuses, Université
Catholique de Lille, F-59160 Lille, France

^3^ Service de Maladies Infectieuses, Centre Hospitalier Métropole Savoie, F-73000 Chambéry, France;
emmanuel.forestier@ch-metropole-savoie.fr

^4^ Clinique Universitaire de Médecine Gériatrique, Centre Hospitalier Universitaire de Grenoble-Alpes,
GREPI EA7408 Université Grenoble-Alpes, F-38000 Grenoble, France; ggavazzi@chu-grenoble.fr

^5^ CHU Bordeaux, Pôle de Gérontologie Clinique, University of Bordeaux, INSERM 1312 BRIC,
F-33000 Bordeaux, France; claire.roubaud@chu-bordeaux.fr

^6^ Service de Maladies Infectieuses, CH Roubaix, F-59056 Roubaix, France; rozenn.hequette@ch-roubaix.fr

^7^ Service Universitaire de Maladies Infectieuses et Tropicales, Hôpital Gustave Dron, F-59200 Tourcoing, France; salfandari@ch-tourcoing.fr (S.A.); esenneville@ch-tourcoing.fr (E.S.)

^8^ Service de Maladies Infectieuses et Tropicales, Centre Hospitalier de Perpignan, F-66000 Perpignan, France; hugues.aumaitre@ch-perpignan.fr

^9^ Infectious Diseases Department, University Hospital of Saint-Etienne, GIMAP (EA 3064), F-42055 Cedex 02 Saint-Etienne, , France; elisabeth.botelho-nevers@chu-st-etienne.fr

^10^ University of Saint-Etienne, Faculty of Medicine of Saint-Etienne, F-42023 Cedex 02 Saint-Etienne, France

^11^ University of Lyon, F-69000 Lyon, France

^12^ Service de Maladies Infectieuses et Tropicales, Hôpital Intercommunal de Villeneuve-Saint-Georges,
F-94190 Villeneuve-Saint-Georges, France; pauline.caraux-paz@chiv.fr

^13^ CHRU-Nancy, Infectious Diseases Department, F-54000 Nancy, France; a.charmillon@chru-nancy.fr

^14^ Grand Est Antibiotic Stewardship Network Coordinator, AntibioEst, F-54000 Nancy, France

^15^ Service de Maladies Infectieuses et Tropicales, Hôpital de Melun, F-77000 Melun, France;
sylvain.diamantis@ghsif.fr

^16^ Unité de Recherche DYNAMIC, Université Paris-Est Créteil, F-94000 Créteil, France

^17^ Court Séjour Gériatrique Aigu, Centre Hospitalier Alès-Cévennes, F-30100 Alès, France; tfraisse@yahoo.fr

^18^ Service des Maladies Infectieuses et Tropicales, CHRU de Brest, F-29609 Brest Cedex, France; pierre.gazeau@chu-brest.fr

^19^ Department of Internal Medicine, Infectious Diseases and Clinical Immunology, University Hospital of Reims, F-51100 Reims, France; mhentzien@chu-reims.fr

^20^ EA3797-Viellissement Fragilité, Reims Champagne Ardennes University, F-51100 Reims, France

^21^ AGIR UR 4294, University Picardie Jules Verne, F-80000 Amiens, France; lanoix.jean-philippe@chu-amiens.fr

^22^ Department of Infectious Diseases, Amiens University Hospital, F-80000 Amiens, France

^23^ Pôle de Gériatrie, CHU Poitiers, Université Poitiers, F-86000 Poitiers, France

^24^ Centre d’Investigation Clinique CIC 1402, INSERM CHU Poitiers, Université Poitiers, F-86000 Poitiers, France; marc.paccalin@chu-poitiers.fr

^25^ Médecine Interne et Maladies Infectieuses, Hôpitaux du Pays du Mont Blanc, F-74700 Sallanches, France

^26^ Physiopathologie et Epidémiologie Cérébro-Cardiovasculaires, Université de Bourgogne, F-21000 Dijon, France;
aputot@ch-sallanches-chamonix.fr

^27^ Department of Infectious Diseases, Strasbourg University Hospital, F-67000 Strasbourg, France;
yvon.ruch@chru-strasbourg.fr

***** Correspondence: baclet.nicolas@ghicl.net; Tel.: +33-320-626-969; Fax: +33-320-626-881

^†^ Membership of the GInGer is provided in the Acknowledgments.

**Supplementary Data S4**

Characteristics of the participants who completed the entire Delphi survey.

| Characteristics | Participants (n = 128) |
| --- | --- |
| Age (median (range)) | 40 (27, 66) |
| Year of MD/PharmD thesis (median (range)) | 2010 (1982, 2021) |
| Female sex | 59 (46.1%) |
| Type of hospital |  |
| General hospital | 49 (38.3%) |
| University hospital | 79 (61.7%) |
| Antimicrobial stewardship activity | 72 (56.2%) |
| Membership of an antibiotic committee | 68 (53.1%) |
| Membership of a learned society’s antibiotic working group | 36 (28.1%) |
| Membership of public health authority dealing with antibiotic use | 10 (7.8%) |
| Medical specialty |  |
| Infectious diseases | 59 (46.1%) |
| Geriatrics | 45 (35.2%) |
| Other (hospital pharmacists, microbiologists, infection control practitioners, general practitioners, neurologists, etc.) | 24 (18.8%) |
